# Lipid composition and antioxidant activities of some underused wild plants seeds from Burundi

**DOI:** 10.1002/fsn3.1969

**Published:** 2020-11-01

**Authors:** Jonathan Niyukuri, Jihane Raiti, Vestine Ntakarutimana, Abdellatif Hafidi

**Affiliations:** ^1^ Food Sciences Laboratory Department of Biology Faculty of Sciences Semlalia Cadi Ayyad University Marrakech Morocco; ^2^ Department of Food Science and Technology Faculty of Agronomy and Bioengineering University of Burundi Bujumbura Burundi; ^3^ Food Science and Technology Research Center (CRSTA) University of Burundi Bujumbura Burundi; ^4^ Department of Chemistry Faculty of Science University of Burundi Bujumbura Burundi; ^5^ Research Center in Natural Sciences and the Environment (CRSNE) University of Burundi Bujumbura Burundi

**Keywords:** antioxidant activity, fatty acids, phenolic content, phytosterols, wild species

## Abstract

Fatty acids, phytosterols, total phenolic content, and radical‐scavenging activity were determined in seed oils of 12 wild plants from natural ecosystems in Burundi. Among the 13 fatty acids identified, palmitic, oleic, linoleic, and stearic acids were found predominant throughout all oils, except *Parinari curatellifolia* oil which showed a high amount of erucic acid (58.41% ± 0.77). The most dominant sterol was found to be β‐sitosterol in all oils and was followed by stigmasterol in 8 kinds of oils and campesterol in 3 kinds of oils. The highest total phenolic contents were observed in *P. curatellifolia, Tephrosia vogelii,* and *Uvaria angolensis* oils, with, respectively, 2.16 ± 0.26, 1.43 ± 0.33, and 1.27 ± 0.39 mg gallic acid equivalent/g oil. Some of these oils exhibited a higher ability to scavenge DPPH radicals. The antioxidant capacity of 8 species ranged from 1.18 to 18.08 mmol acid ascorbic equivalent/g oil. Based on these findings, such oils could be used in different domains such as food, cosmetic, pharmaceutical, and lipochemistry.

## Graphical abstract

This study showed 14 species of wild plants that can provide oils with a wide variety of chemical compositions. They have been characterized by high contents of polyunsaturated fatty acids while they exhibit several positive health effects. These results suggest the important utility in various domain such as energy, cosmetics and especially the diversification of edible oils.

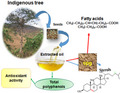

## 1 INTRODUCTION

Vegetable oils are raw materials, with a wide range of use in our life. In addition to the steadily growing international demand in response to the continuous increase of the world population, new domains of use of oils appear from day to day. The projection performed by Carlsson et al., (2011) showed that global vegetable oils requirements, estimated at 150 million metric tons in 2010, will increase to 400 million metric tons in 2030. The extension of the domain of use of vegetable oils goes hand in hand with the different discoveries related to its different chemical compositions and physicochemical properties. Jatropha oil, which has properties like viscosity, flashpoint 65, and ignition point closely approaching those of diesel, is reported to be used as an interesting alternative in diesel engines (Thapa et al., 2017). Furthermore, plant oils are used as functional foods with interesting properties, including antioxidant, anticarcinogenic, and photo‐protector (Antignac et al., 2011). Regarding lipid compounds, investigations have already specified the individual importance of many molecules. Palmitic and stearic acids are reported to have capacity to stimulate melanogenesis, while oleic and linoleic acids and vitamin E have a good antioxidant activity (Chaikul et al., 2017). The epoxides obtained from the oleic fatty acids are used in oleochemistry mainly as PVC stabilizers (Bornscheuer, 2006), and omega‐3 fatty acids are very renowned due to their preventive capacities for various disease. Furthermore, many studies have reported that oils are major sources of tocopherols (Górnas´, 2015; Soliven, 2014; Górnaś et al., 2017).The consumption of additional phytosterols at a maximum effective dose of 2 g/day significantly reduced LDL levels from 9% to 14% (Kritchevsky and Chen, 2005). Phytosterols are also involved in regulation of membrane fluidity and thus influence membrane properties, functions, and structure (Korber et al., 2017; Ostlund, 2002), decrease cholesterol accumulation, risks of coronary heart disease and contribute to inhibiting the absorption of intestinal cholesterol, including recirculating endogenous biliary cholesterol (Ostlund, 2002). Many countries have already started to value the underused resources of their plants to produce new oils. Morocco has studied and developed the argan plant which has become very known worldwide (Farres et al., 2019). Burundi has an important plant resources estimated at 3,125 species including 70 endemics (MEEATU (Ministère de l’Eau, de l’Environnement, de l’Aménagement du T. et de l’Urbanisme), 2013). However, most of them are not yet studied. Furthermore, Burundi's oil production covers only 20% of the domestic consummation (MINAGRI, 2008). The diversification of the natural sources of vegetable oils will help to reach our national demand and also will lead to a sustainable valorization and preservation of the biodiversity. The present study was carried out on twelve wild species; *Entada abyssinica* Steudel ex A. Rich*, Annona senegalensis* Pers (Annonaceae), *Brachystegia longifolia* Benth (Fabaceae)*, Caesalpinia decapetala* (Roth) Alston (Caesalpiniaceae)*, Dodonaea viscose* Jacq (Sapindaceae)*, Entada abyssinica* A. Rich. (Mimosaceae), *Ipomoea involucrate* P. Beauv (Convolvulaceae)*, Myrianthus arboreus* Beauv. (Cecropiaceae)*, Maesopsis eminii* Engl (Rhamnaceae)*, Parinari curatellifolia* Planch. Ex Benth (Chrysobalanaceae)*, Sterculia tragacantha* Lindl (Malvaceae)*, Tephrosia vogelii* Hook.f*. (*Fabaceae)*, Uvaria angolensis* Welw. ex Oliv (Annonaceae); from natural ecosystems of Burundi. These species, locally used as firewood, edible fruit, timber, and traditional medicines, have been reported to have potentialities to produce oil (Niyukuri et al., 2019). However, there are no scientific data on their oil chemical compositions. For this purpose, our investigation aims to characterize the lipid profile of these wild species, total phenolic content in the oils, and assessment of antioxidant capacity from oils samples measured with 2,2‐diphenyl‐1‐picrylhdrazyl (DPPH) assay.

## 2 MATERIALS AND METHODS

### 2.1 Plant material

Fruits were sampled from May to December 2018 according to the period of fruit ripening of the plant. The harvest was conducted in five ecoclimatic zones, namely: the plain (775–1000 m of altitude), the foothills (1000–1500 m of altitude), the high mountains (1500–2600 m), the central trays (1,400 –2000 m of altitude), and the depressions (1200–1500 m of altitude) (Table 1). The sampled species were *E. abyssinica, A. senegalensis, B. longifolia, C. decapetala, D. viscosa, I. involucrata, M. arboreus, M. eminii, P. curatellifolia, S. tragacantha, T. vogelii,* and *U. angolensis*. Each species was harvested in three different sites. The identification of the species was performed at the herbarium of the University of Burundi and the herbarium of the Burundian Office for the Protection of the Environment (OBPE). After drying, the seeds were hulled manually. Then, oil extraction was performed with hexane as solvent in a Soxhlet apparatus under reflux for 8 hr. The solvent was evaporated under reduced pressure. The acidity was determined according to the ISO 660:2009 method (ISO, 18609, 2000).

**Table 1:** Seeds sampling

Plant species	Vernacular name	Sampling period (2018)		P	FH	HM	CT	D
*Annona senegalensis*	Umukanda, Umutobe		October to November		1		1	1
*Brachystegia longifolia*	Ingongo		October to November	1	2			
*Caesalpinia decapetala*	Uruzira, Umubambangwe		July to October			1	1	1
*Dodonaea viscosa*	Umusasa		July to October			1	1	1
*Entada abyssinica*	umusange		July to October		1		1	1
*Ipomoea involucrata*	Umurandaranda		July to October		1	1		1
*Maesopsis eminii*	Umuhumura, Indunga		July to September		2			1
*Myrianthus arboreus*	Umwufe		October to November	2	1			
*Parinari curatellifolia*	Umunazi		August to October		1		1	1
*Sterculia tragacantha*	Umutakataka		July to September		2			1
*Tephrosia vogelii*	Ntibuhunwa		June to August		1		1	1
*Uvaria angolensis*	Umubungo		July to October		2		1	

Note

P: Plain, FH: foothills of Mumirwa, HM: high mountains, CT: central trays, D: depressions, 1: the species is sampled on the same site of an ecoclimatic zone, 2: a species is sampled at two (communes) on the same ecoclimatic zone.

### 2.2 Chemicals

All chemicals and solvents were of analytical grade and used without further purification. Chloroform, ethanol, methanol, n‐hexane, anhydrous magnesium sulfate, sodium carbonate, DPPH (2,2‐diphenyl‐1‐picrylhydrazyl), Folin–Ciocalteu reagent, gallic acid, ascorbic acid, potassium hydroxide, sodium hydroxide, sodium sulfate anhydride, anhydrous pyridine, Hexamethyldisilazane, trimethylchlorosilane, chlorohydrin acid, isooctane, petroleum ether, sodium chloride, diethyl ether, and ethyl acetate were purchased from Sigma Aldrich Chemical Co.

### 2.3 Fatty acid analysis

Fatty acid methyl esters (FAMEs) were prepared in two ways according to the oil acidity. Oils with an acidity ˂ 3,3% were methyled following alkaline conditions as described by Bannon et al. (1981) method, while oils with a free fatty acid content ≥ 3,3% were performed as described by Jham et al., (1982). A Shimadzu GC‐2010 Plus equipped with a flame ionization detector and Capillary column (30 m × 0.25 mm; film thickness 0.25 µm) was used. Column temperature, 180°C; injector temperature, 225°C; detector temperature, 250°C; injection volume, 1 μl; carrier gas, nitrogen; and flow rate, 1 ml/min. Total running time was 20 min. Identification of individual fatty acids was performed by comparing their retention times with a certified fatty acid methyl esters mix and quantified as percentage of total fatty acids.

### 2.4 Sterols analysis

The extraction of the unsaponifiable material was carried out as described by ISO 18,609:2000 (International Organization for Standardization– ISO, 2000). An internal standard, 5α‐cholestane, was used for sterols quantification. The sterol fraction from the unsaponifiable matter was purified by thin‐layer chromatography (TLC) on 20 × 20 cm silica gel, 0.25 mm thickness of layer using hexane/diethyl ether (1/1:v/v). The extracted sterol fraction from the TLC has been subjected to silylation. Thus, Trimethylsilyl (TMS) ether derivatives of sterols were prepared according to the method described by Savage et al. (1997) for further analyses in a Shimadzu GC‐2010 Plus equipped with a flame ionization detector and column SUPELCO SAC‐5 (30 m × 0.25 mm × 0.25 µm). A sample of 1.0 µl was injected in a split mode. The column was held at isocratic temperature of 280°C during 35 min. The detector temperature was set at 300°C. Hydrogen was used as a carrier gas at a flow rate of 0.35 ml/min. Sterols were identified by comparing their retention times relative to 5α‐cholestane, and results were expressed as mg/kg of oil.

### 2.5 Methanol/water extracts preparation

Methanolic/water (80/20:V/V) extraction was performed following the method described by Kalantzakis et al. (2006). A sample of oil (1 g) was dissolved with 5 ml of n‐hexane, and then, 5 ml of methanol/water was added. The mixture was vigorously stirred by vortexing for 10 s and centrifuged at 3,500 *rpm* for 10 min. The methanol/water phase was separated from the lipid phase using Pasteur pipette, and the residue was extracted twice with a new portion of methanol/water (2 × 5 ml). The methanol/water phases were combined and evaporated under reduced pressure using a rotary evaporator at 45°C.

### 2.6 Total phenolic content determination

Total phenolic contents of methanol/water extracts were determined using the Folin–Ciocalteu reagent (Singleton & Rossi, 1965) with minor modification. The methanol/water extract (0.5 ml) was diluted with water (4 ml), and then, the Folin–Ciocalteu's reagent (0.5 ml) was added. After 3 min, 1 ml of a sodium carbonate solution (1.9 M) was added and filled up to 10 ml with water. The samples were left to stand in darkness for 60 min, and then, the absorbance was measured at 760 nm using a UV/Vis spectrophotometer. Total phenolic content in each sample was determined using a standard curve prepared using gallic acid. The results were expressed as milligrams of gallic acid per gram of oil. However, the Folin–Ciocalteu reagent method used does not explain exactly polyphenols contents, as it can react with other compounds. But this method is widely used to rapidly appreciate of the phenolic compounds amounts after an extraction with methanol and a purification with the ethyl acetate. In such extracts, the phenolics were assumed to be dominant over other possible reacting compounds. The current trend is that the Folin–Ciocalteu reagent measures sample reducing capacity assay (Górnaś et al., 2015; Rior, 2005).

### 2.7 Measurement of antioxidant activity‐DPPH assay

The antioxidant activity of the methanol/water extracts and seed oil samples was determined using DPPH radicals as described by Kozłowska et al. (2016), with some modifications. 0.5 ml of each methanolic extract of seed oils was diluted with 3.25 ml of methanol, and then, 0.25 ml of 1 mM methanolic solution of DPPH was added. The mixture was vigorously mixed for 10 s in a vortex apparatus and kept in darkness for 10 min. The absorbance was measured at 515 nm using a UV/Vis spectrophotometer. The results were expressed as millimoles ascorbic acid equivalent antioxidant capacity using an ascorbic acid calibration curve (mmol AAE/g oil). In order to determine the antiradical activity in seed oil samples, 50 mg of each oil was dissolved in 3.0 ml of ethyl acetate. Then, 1 ml of an oil solution was diluted with ethyl acetate (2.75 ml) and 0.25 ml of a freshly prepared DPPH solution (1 mM) was added. The samples were vigorously mixed for 10 s in a vortex, and after 20 min, the absorbance was measured at 515 nm using a UV/Vis spectrophotometer. The result was expressed as millimoles ascorbic acid equivalent antioxidant capacity using an ascorbic acid calibration curve (mmol AAE/g oil).

### 2.8 Statistical analysis

Data analysis was performed using IBM SPSS statistic 20. Results were analyzed using one‐way analysis of variance (ANOVA) followed by Duncan's multiple comparison test. Correlation between various parameters was also computed. Significance was determined at *p* < .05 level, and the results were expressed as mean values ± standard error (SE). Hierarchical clustering analysis (HCA) was applied to classify the oils according to their chemical composition.

## 3 RESULTS AND DISCUSSION

### 3.1 Oil contents

The oil contents have been found to be economically exploitable. Nine species showed contents greater than 15% where the largest reached was 63% extracted from *P. curatellifolia* species. The contents of all of the oils provided by these species studied are almost similar to those found by Niyukuri et al. (2019). However, small differences of the order of 1%–2% have been recorded in some species. The oils contents having been sufficiently discussed in previous study, this paper was focused on their chemical compositions.

### 3.2 Fatty acid profiles

#### 3.2.1 Fatty acid composition

Of the thirteen FAs detected (myristic, C14: 0; palmitic, C16: 0; palmitoleic, C16: 1n‐7; stearic, C18: 0; oleic, C18: 1n‐9; linoleic, C18: 2 n‐6; linolenic, C18: 3 n‐3; arachidic, C20: 0; gadoleic acid, C20: 1 n‐9; behenic, C 22:0; erucic, C22: 1 n‐9; lignoceric, C24: 0; selacholeic, C24: 1), six FAs (C16: 0, C18: 0, C18: 1n‐9, C18: 2 n‐6, C18: 3 n‐3 et C20: 1 n‐9) were present in all type of oils as depicted in Table 2. However, these fatty acids prevailed in oils at very different concentrations. While C14:0; C16: 1n‐7; C20: 0,C20: 1 n‐9, and C24: 0 did not exceed 5%, C18: 2n‐6 reached 89% in the oil of *M. arboreus*. C16: 0; C18: 1n‐9 and C18: 2 n‐6 were found to be the major fatty acid in almost all oil. Thus, the great disparity in the distribution of fatty acids led us to distinguish 3 different classes of oils considering number of the fatty acids contributing to a minimum of 70% of the total content in the oil. The first group was found to reach 70% with a single fatty acid while the second with two fatty acids. In the third group, it necessitated 3 fatty acids to reach 70% of the total content.

**Table 2:** Relative Fatty Acid (FA) composition and oil content of vegetable oils extracted from wild seed plants harvested in Burundi natural ecosystem

FA (%)	Sample of seedoils					
	AS	BL	CD	DV	EA	II
C14: 0	0.18 ± 0.03^b^	3.96 ± 0.02^a^	–	–	–	0.10 ± 0.00^b^
C16: 0	12.17 ± 0.26^a^	18.78 + 0.90^f^	6.16 ± 0.12^e^	9.80 ± 0.13^c^	8.00 ± 0.03^d^	11.96 ± 0.42^e^
C16: 1n‐7	0.33 ± 0.01^ab^	–		0.41 ± 0.00^a^	0.14 ± 0.00^ab^	0.75 ± 0.01^a^
C18: 0	6.09 ± 0.26^b^	22.75 ± 0.28^a^	4.58 ± 0.02^bc^	2.29 ± 0.06^de^	16.40 ± 6.00^b^	2.53 ± 0.03^cde^
C18: 1n‐9	36.85 ± 0.14^b^	39.17 ± 0.38^b^	14.65 ± 0.05^ef^	18.53 ± 0.18^fg^	47.92 ± 0.48^a^	31.82 ± 0.02^bc^
C18: 2 *n*‐6	40.95 ± 0.33^d^	1.84 ± 0.18^g^	73.37 ± 0.09^b^	60.05 ± 0.77^b^	31.01 ± 0.09^e^	44.52 ± 0.25^d^
C18: 3 *n*‐3	1.54 ± 0.09^bcd^	1.24 ± 0.10^bcd^	0.38 ± 0.01^cd^	0.82 ± 0.09^cd^	0.68 ± 0.27^cd^	1.64 ± 0.01^bc^
C20: 0	0.73 ± 0.10^e^	0.30 ± 0.00^g^	–	3.41 ± 0.08^a^	1.94 ± 0.03^c^	1.22 ± 0.06^d^
C20: 1 *n*‐9	0.34 ± 0.08^e^	5.00 ± 0.51^a^	0.14 ± 0.00^e^	1.42 ± 0.36^bcd^	0.76 ± 0.00^de^	1.07 ± 0.04^cde^
C22: 0	–	0.41 ± 0.00^d^	–	1.37 ± 0.08^c^	1.31 ± 0.01^c^	2.65 ± 0.30^b^
C22: 1 *n*‐9	–	6.21 ± 0.58^b^	0.14 ± 0.00^d^	–	–	–
C24: 0	–	–	0.13 ± 0.01^c^	–	2.42 ± 0.04^a^	1.67 ± 0.19^b^
C24: 1	0.25 ± 0.11^c^	–	–	–	–	–
∑ SFAs	19.17 ± 0.21^e^	45.18 ± 0.54^c^	11.28 ± 0.09^h^	16.87 ± 0.20^g^	30.07 ± 0.13^e^	20.13 ± 0.35^e^
∑ MUFAs	37.77 ± 0.16^c^	50.38 ± 0.97^b^	14.93 ± 0.47^g^	19.95 ± 0.55^f^	48.82 ± 0.66^f b^	33.64 ± 0.48^d^
∑ PUFAs	42.49 ± 0.32^e^	3.08 ± 0.54^i^	73.75 ± 0.24^b^	60.87 ± 0.96^c^	31.69 ± 0.52^f^	46.16 ± 0.34^d^
PUFA/SFA	2.216 ± 0.03^def^	0.11 ± 0.01^g^	6.3 ± 0.07^b^	3.60 ± 0.01^c^	1.05 ± 0.01^ef^	2.29 ± 0.03^cde^
HC (%)	29.67 ± 1.13^c^	8.15 ± 0.43^e^	17.30 ± 1.73^de^	16.71 ± 0.90^def^	15.88 ± 3.01^def^	12.12 ± 1.95^def^

FA (%)	Sample of seedoils					
	MA	ME	PC	ST	TV	UA
C14: 0	–	–	–	–	–	–
C16: 0	1.17 ± 0.08^g^	7.54 ± 0.39^d^	4.73 ± 0.06^f^	29.83 ± 0.72^a^	11.59 ± 0.79^b^	7.55 ± 0.01^d^
C16: 1n‐7	0.08 ± 0.01^b^	0.11 ± 0.00^ab^	–	–	0.14 ± 0.01^ab^	0.38 ± 0.00^ab^
C18: 0	1.16 ± 0.04^e^	21.48 ± 2.91^a^	4.91 ± 0.02^b^	3.80 ± 0.49^bcd^	4.65 ± 0.41^bc^	4.76 ± 1.59^b^
C18: 1n‐9	5.43 ± 0.24^g^	39.42 ± 0.25^b^	17.63 ± 0.11ef	17.67 ± 0.32^def^	20.48 ± 4.72^de^	23.99 ± 3.33^cd^
C18: 2 *n*‐6	89.54 ± 1.57^a^	23.09 ± 3.04^f^	9.21 ± 0.02^g^	23.21 ± 0.16^f^	45.98 ± 0.36^d^	57.72 ± 2.15^c^
C18: 3 *n*‐3	0.23 ± 0.03^e^	2.46 ± 0.06^b^	–	–	9.99 ± 0.02^a^	1.03 ± 0.02^cd^
C20: 0	–	2.60 ± 0.16^b^	0.34 ± 0.01^f^	–	1.94 ± 0.08^c^	0.67 ± 0.01^e^
C20: 1 *n*‐9	0.34 ± 0.19^e^	1.72 ± 0.03^bc^	1.45 ± 0.81^f^	2.28 ± 0.12^b^	0.62 ± 0.04^de^	0.67 ± 0.13^e^
C22: 0	0.20 ± 0.01^d^	0.64 ± 0.04^cd^	–	4.75 ± 0.99^a^	5.76 ± 0.80^a^	0.39 ± 0.01^cd^
C22: 1 *n*‐9	–	–	58.41 ± 0.77^a^	2.06 ± 0.01^c^	0.20 ± 0.02^d^	–
C24: 0	–	0.34 ± 0.02^c^	–	–	1.55 ± 0.22^b^	–
C24: 1	1.05 ± 0.16^b^	–	–	6.68 ± 0.07^a^	–	–
∑ SFAs	2.53 ± 0.29^i^	32.6 ± 3.74^b^	9.98 ± 0.11^h^	38.38 ± 0.44^a^	25.49 ± 1.33^d^	13.37 ± 2.54f
∑ MUFAs	6.9 ± 0.89^h^	41.25 ± 0.31^c^	77.49 ± 0.95^a^	28.69 ± 0.35^f^	21.44 ± 6.18^f^	25.04 ± 3.67^e^
∑ PUFAs	89.77 ± 2.11^a^	25.55 ± 3.19^g^	9.21 ± 0.02^h^	23.21 ± 2.11^f^	55.97 ± 3.14^de^	58.75 ± 2.51^c^
PUFA/SFA	35.48 ± 3.38^a^	0.78 ± 0.10^ef^	0.92 ± 0.00^ef^	0.60 ± 0.05^et^	2.19 ± 0.08^ef^	4.39 ± 1.03^cd^
HC (%)	51.07 ± 8.60^b^	47.01 ± 6.05^b^	63.25 ± 9.09^a^	33.78 ± 4.86^c^	10.11 ± 0.50^ef^	20.11 ± 0.30^d^

Note

AS, Annona senegalensis; BL, Brachystegia longifolia; CD, Caesalpinia decapetala. DV, Dodonaea viscosa; EA, Entada abyssinica; HC, oil contents; II, Ipomoea involucrate; MA, Myrianthus arboreus; ME, Maesopsis eminii; PC, Parinari curatellifolia; ST, Sterculia tragacantha; TV, Tephrosia vogelii; UA, Uvaria angolensis.

Data represent means ± *SD* (standard deviation) (*n* = 3). –: not identified. FA: fatty acid (C14: 0, myristic; C16: 0, palmitic acid; C16: 1n‐7, palmitoleic; C18: 0,stearic; C18: 1n‐9, oleic; C18: 2 *n*‐6, linoleic; C18: 3 *n*‐3, linolenic; C20: 0, arachidic; C20: 1 *n*‐9, gadoleic; C22: 0, behenic; C22: 1 *n*‐9, erucic; C24: 0, lignoceric; C24: 1, selacholeic).∑SFA: sum of saturated fatty acids; ∑MUFA: sum of monounsaturated fatty acids; ∑PUFA: sum of polyunsaturated fatty acids. Values with different superscript letters ^(a‐i)^ within each row are significantly different at *p* < .05.

##### Oils with one extra major fatty acid (Linoleic oil)

*M. arboreus* oil is exceptionally high in linoleic acid (89.54% ± 1.57), whereas 8 other FAs shared the remaining portion of 10.46%. Also for *C. decapetala* oil, the linoleic acid occupied 73.37% ± 0.09 while the following important contributor was found to be oleic acid (14.65% ± 0.05) and six other FAs identified accounted for 12%. Such linoleic acid content is rarely encountered in conventional oils. In these latters, the high contents reached have been reported in sunflower oil (61%) (Wang et al., 2019). So, *M. arboreus* and *C. decapetala* oils may be of a great importance to improve the nutritional quality of other oils with low polyunsaturated fatty acids contents like palm oil.

##### Oils with two major fatty acids

This class consisted of oils with two fatty acids totalizing at least 70% of the FAs content. It is a large group that has combined more than half of the studied oils (*A. senegalensis, D. viscosa*, *E. abyssinica*, *I. involucrate, P. curatellifolia, and U. angolensis*) in which we can distinguish two subclasses: Linoleic–Oleic and Erucic oils. Such denomination were reported for other common oils: oleic sunflower oil (De Figueiredo et al., 2019), oleic rapeseed oil, and erucic oilseed rape (Zanetti et al., 2009).

**Linoleic–Oleic oils:** In this subclass where linoleic acid and oleic acids are predominant gathered oils of *A. senegalensis*, *D. viscosa, I. involucrate, U. angolensis, and E. abyssinica*. Some of these oils have showed quite similar FA profiles. Thus, comparable FAs profiles were determined in *A. senegalensis, U. angolensis* oils. This may be because they belong to the same family of the Annonaceae. Linoleic and oleic acid proportions were 40.95% ± 0.33 and 36.85% ± 0.14 80, respectively, for *A. senegalensis,* and 57.72% ± 2.15 and 23.99% ± 3.33 for *U. angolensis*. The following important contributor was palmitic acid at 12.17% ± 0.26 in *A. senegalensis* oil and 7.55% ± 0.01 in *U. angolensis* oil, while the other six fatty acids have contents less than 5%. *A. senegalensis* oil profile was found to be quite similar to that reported in *Jatropha curcas* (Kumar & Das, 2018). Since *Jatropha curcas* oil is an excellent biodiesel (Chaudhary et al., 2018), *A. senegalensis* oil may have also high probability of being a source of biofuel.

Interesting contents of linoleic and oleic acid have been also detected in *D. viscosa oil*. They represented 60.05% ± 0.77 and 18.53% ± 0.18, respectively. The six other identified FAs did not exceed 21%. Furthermore, *D. viscose* oil has been found to be a very good source of linoleic acid in this subclass and class.

In *I. involucrate* oil, 11 FAs were detected. The main components were found to be linoleic acid (44.52% ± 0.25) and oleic acid (31.82% ± 0.02).The palmitic acid represented 11.9%, while the other eight fatty acids ranged from 0.10% to 2.56%. A similar trend of fatty acid composition has been reported in Japanese quince seed oil (Górnaś et al., 2013).

*E. abyssinica* showed an oil composition with 10 FAs. Oleic and linoleic acids contributed at 47.92% ± 0.48 and 31.01% ± 0.09, respectively, and the lowest content FA detected was palmitoleic acid (0.14% ± 0.00).

**Erucic oils**: *P. curatellifolia* oil was distinguished from all the other oils studied by its high amounts of the erucic acid (58.41% ± 0.77).This content is much higher than that found in erucic rapeseed (Zanetti et al., 2009). According to (Merrien et al., 2012), oils with such content of erucic acids could be used as lubricants, detergents, soaps, erucamide nylon (polyethylene, paints, etc), and cosmetics. The second main contributor in *P. curatellifolia* oil was oleic acid (17.63% ± 0.11), while other 5 FAs did not exceed 10%.

##### Oils with three major fatty acids

In this class, 4 species (*B. longifolia, M. eminii, S. tragacantha*, and *T. vogelii*) showed oils in which three FAs contributed to more than 70% of the total FA content. Among this category, three subclasses can be distinguished.

**Subclass 1:** In this subclass, we found *S. tragacantha* and *T. vogelii* oils characterized by three major fatty acids (palmitic, oleic, and linoleic). However, their chemical profiles are different. *T. vogelii* oil was found to contain eleven fatty acids, whereas *S. tragacantha* had eight fatty acids. The latter was also particular due to its relatively high palmitic acid content (29.83% oil). Nevertheless, it was found to be much lower than that reported in oil palm. The second contributor in *S. tragacantha* oil was linoleic acid (23.21% ± 0.16) followed by oleic acid (17.67% ± 0.32). Regarding *T. vogelii* oil, the fatty acid with the highest content was found to be linoleic (45.98% ± 0.36), and oleic acid with 20.48% ± 4.72 was the second while palmitic acid (11.59% ± 0.79) was the third. Compared to some commonly used oils, chemical profile of soybean oil (Zanetti et al., 2009) was very close to that of *T. vogelii* oil.

**Subclass 2:** Only oil from *B. longifolia* was found in this group in which the three main fatty acids totalizing more than 70% of the whole FAs content were found to be oleic acid (39.17% ± 0.38), stearic acid (22.75% ± 0.28), and palmitic acid (18.78% ± 0.90). All the other seven fatty acids did not exceed 7% among them two minor fatty acids (arachidic and behenic) representing less than 0.5% each.

**Subclass 3:** It is also represented by a single specie: *M. eminii* oil. Thus, out of 10 FAs detected, oleic, linoleic, and stearic acid were represented at 39.42% ± 0.25, 23.09% ± 3.04, and 21.48% ± 2.91, respectively. The lowest content found was 0.11% of palmitoleic acid.

#### 3.2.2 Composition of main classes fatty acids

The main classes of fatty acids in the studied oils (total saturated fatty acids (SFAs), monounsaturated fatty acids (MUFAs), and polyunsaturated fatty acids (PUFAs)) are summarized in Table 2. Overall, SFAs were detected in all oils studied but in low contents compared to MUFAs and PUFAs. *S. tragacantha* oil is characterized by a high content of SFA (38.4%) while for other oils SFA ranged only from 2.5% to 30%. The saturated fatty acids were found to be palmitic (C16:0), stearic (C18:0), and myristic acid (C14:0) which is detected as traces in some oils. Concerning MUFAs, they were found to be the second contributors for total FA contents. Oils extracted from 4 species were found to have high MUFAs contents. Their MUFAs amounts were significantly different (*p* < .05) and ranged in the following order: *P. curatellifolia* (77%)> *B. longifolia* (50%)> *E. abyssinica* (48%)> *M. eminii* oils (41%). Among the 5 MUFAs detected, oleic acid (C18:1) was the main representant. Its content exceeded 78.69% of total MUFAs in all oils except in *S. tragacantha* oil which represented 61.58% of total MUFAs and in *P. curatellifolia* in which, the erucic acid (C22:1) represented 77.37% of total MUFAs. PUFAs were the most prevalent in the studied oils. However, they consisted only in two major fatty acids: linoleic (C18:2 n‐6) and α‐linolenic acid (C18:3 n‐3). The highest PUFA contents reached 89.7, 73.7, 60.8, 58.7, and 55.9% and were identified in *M. arboreus, C. decapetala, D. viscosa*, *U. angolensis*, *and T. vogelii* oil, respectively. According to these findings, these oils would make a significant contribution in improving human health especially in Burundi where Palm oil is the major edible oil. PUFAs are reported to have the potential to prevent cardiovascular, inflammatory, and cancer diseases and to reduce the risk of atherosclerotic plaque (Nelson et al., 2017). In addition, (Mason et al., 2018) reported that PUFAs inhibited endothelia dysfunction due to the hyperglycemia, oxidative stress, and dyslipidemia.

#### 3.2.3 Hierarchical clustering analysis classification

**Figure 1:**
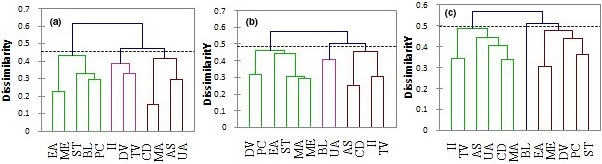
Cluster analyses of twelve vegetable oils using the fatty acids (A), the phytosterols *(B)*, and combined data (C). PC, *Parinari curatellifolia;* MA, *Myrianthus arboreus;* ME, *Maesopsis eminii;* ST, *Sterculia tragacantha;* AS, *Annona senegalensis;* UA, *Uvaria angolensis;* CD, *Caesalpinia decapetala*; DV; *Dodonaea viscosa;* EA, *Entada abyssinica;* II, *Ipomoea involucrata;* TV, *Tephrosia vogelii;* BL, *Brachystegia longifolia*

Considering the contribution of all fatty acids in their respective oils, the *Hierarchical clustering analysis* (HCA) test allowed us to classify oils in comparable groups (oils with almost the same or close profiles) (Figure 1a). At the similarity distance of 0.2, the segregation produced three clusters (1, 2, and 3). Cluster 1 consisted of two species (*P. curatellifolia* and *B. longifolia*). They were brought closer together by their high MUFAs contents: 77.49 > 50.38% (*p* < .05), respectively, for *P. curatellifolia* and *B. longifolia* oil. Cluster 2 was consisting of only *Sterculia tragacantha* oil, which was distinguished from all other oils by its high SFA content (38.38%). Cluster 3 combined nine species. They divided into 2 subclusters. The first subcluster comprised single specie characterized by a high content of PUFAs. It was *M. arboreus* with 89.77% of PUFA, significantly higher than those of other species (*p* < .05). The second subcluster included 8 species. It is suggested that there is more probability to have one fatty acid in these 8 different oils.

### 3.3 Sterols profiles of the studied oils

#### 3.3.1 Sterols compositions

Phytosterols contents in the studied oils are shown in Table 3. Fifteen different phytosterols were identified through the 12 oils. They are as follows: cholesterol, brassicasterol, 24‐methylene‐cholesterol, campesterol, campestanol, stigmasterol, ∆‐7‐campesterol, ∆‐5‐23‐stigmastadiénol, clerosterol, β‐sitosterol, sitostanol, ∆‐5‐avenasterol, ∆‐5‐24‐stigmastadiénol, ∆‐7‐stimasterol, and ∆‐7‐avenasterol. Among them, campesterol, stigmasterol, and β‐sitosterol were found to be the major compounds in all oil samples. Wide variations in total sterol levels were observed throughout the studied oils. Thus, the lowest sterol content was found in *S. tragacantha* oil (2,578 mg/kg of oil), and 7 oils were found to have more than 5 000 mg/kg while the highest content was detected in *B. longifolia* oil (21,630.98 mg/kg). These results remain within the limits reported in common oils such virgin olive oil (3,280 mg/kg), crude borage oil (5,680 mg/kg), expeller pressed sunflower (6,920 mg/kg), canola oil (13,260 mg/kg), corn oil 15,320 mg/kg), and crude evening primrose (21,940 mg/kg) (Phillips et al., 2002).

**Table 3:** Sterol compositions of vegetable oils extracted from wild seed plants harvested in Burundi natural ecosystem

Sterols (mg/kg oil)	Sample of seed oils					
	AS	BL	CD	DV	EA	II
Cholesterol	–	95.50 ± 1.15^a^	59.62 ± 0.94^b^	39.29 ± 1.64^bc^	47.35 ± 4.10^bc^	58.77 ± 0.68^b^
Brassicasterol	–	–	–	13.33 ± 11.55^c^	–	21.33 ± 4.04^bc^
24‐methylene‐cholesterol	–	–	–	–	231.04 ± 1.30^a^	–
campesterol	1,332.60 ± 130.16^b^	2,555.83 ± 1.96^a^	1,040.19 ± 13.35^c^	143.29 ± 1.72^f^	231.00 ± 2.83^e^	384.73 ± 1.70^d^
Campestanol	–	77.55 ± 1.44	–	–	–	–
Stigmasterol	2,133.41 ± 182.97^b^	1,335.14 ± 1.30^d^	1,482.55 ± 11.66^c^	552.09 ± 5.44^e^	599.30 ± 5.35^e^	2,597.77 ± 13.17^a^
∆‐7‐campesterol	–	33.00 ± 1.73^b^	23.00 ± 0.00^d^	99.00 ± 2.65^a^	25.00 ± 4.58^c^	39.00 ± 2.00^ab^
∆‐5, 23‐stigmastadiénol	64.06 ± 6.07^b^	103.34 ± 28.74^a^	53.80 ± 0.92^b^	67.83 ± 2.28^b^	–	–
Clerosterol	–	–	25.72 ± 0.76^b^	–	–	46.59 ± 3.77^b^
β‐sitosterol	6,516.64 ± 189.32^d^	16,564.81 ± 15.65^a^	5,208.00 ± 18.20^e^	2,168.94 ± 10.37^j^	3,914.65 ± 20.81^g^	7,251.42 ± 2.15^c^
Sitostanol	34.60 ± 8.66^e^	645.49 ± 24.21^a^	125.82 ± 1.95^c^	88.63 ± 13.51^d^	134.31 ± 3.67^c^	53.22 ± 28.04^e^
∆−5‐avenasterol	189.29 ± 29.37^e^	54.71 ± 8.29^f^	–	372.88 ± 2.67^b^	33.96 ± 4.17^f^	293.64 ± 47.12^C^
∆‐5‐24‐stigmastadienol	61.93 ± 3.94^b^	26.44 ± 2.59^d^	40.23 ± 0.26^c^	–	65.53 ± 3.36^ab^	41.78 ± 2.62^c^
∆‐7‐stimasterol	–	31.13 ± 1.43^d^	–	208.27 ± 0.75^a^	102.55 ± 2.02^b^	74.52 ± 8.74^c^
∆‐7‐avenasterol	–	–	–	–	–	27.63 ± 1.84^c^
Total sterols	10,332.53 ± 12.72^c^	21,522.94 ± 1,433.21^a^	8,058.93 ± 41.06^d^	3,753.55 ± 94.08^ef^	5,384.69 ± 82.38^e^	10,890.4 ± 1,116.8^c^

Sterols (mg/kg oil)	Sample of seedoils					
	MA	ME	PC	ST	TV	UA
Cholesterol	63.11 ± 9.80^b^	24.83 ± 3.87^c^	96.72 ± 0.56^a^	104.32 ± 53.59^a^	56.74 ± 12.92^b^	108.92 ± 5.40^a^
Brassicasterol	–	–	82.06 ± 0.65^ab^	105.55 ± 49.38^a^	–	–
24‐methylene‐cholesterol	–	40.83 ± 5.92^c^	–	124.83 ± 7.93^b^	–	–
campesterol	150.53 ± 1.23^f^	120.25 ± 5.18^f^	183.81 ± 1.19^ef^	259.26 ± 103.40^e^	862.44 ± 8.96^d^	1,047.90 ± 15.89^c^
Campestanol	–	–	–	–	–	–
Stigmasterol	251.09 ± 31.47^g^	115.63 ± 0.51^h^	599.35 ± 2.52^e^	448.79 ± 59.52^f^	2,610.92 ± 49.13^a^	1,363.00 ± 56.35^d^
∆‐7‐campesterol	–	–	72.33 ± 2.89^a^	–	41.00 ± 6.56^a^	–
∆‐5‐23‐stigmastadiénol	–	–	–	–	–	–
Clerosterol	34.12 ± 8.81^b^	–	–	–	148.51 ± 26.67^a^	–
β‐sitosterol	3,192.18 ± 111.42^h^	3,203.11 ± 45.52^h^	2,323.60 ± 15.94^i^	1,234.93 ± 149.21^k^	13,115.22 ± 122.61^b^	5,026.12 ± 36.42^f^
Sitostanol	26.20 ± 3.10^e^	27.84 ± 8.95^e^	–	146.92 ± 33.13^c^	415.36 ± 31.18^b^	–
∆‐5‐avenasterol	228.80 ± 7.57^d^	214.44 ± 3.19^d^	293.58 ± 4.53^c^	153.49 ± 18.30^e^	522.35 ± 7.22^a^	–
∆‐5‐24‐stigmastadienol	–	17.38 ± 4.23^e^	–	–	70.32 ± 9.33^a^	–
∆‐7‐stimasterol	–	–	383.53 ± 6.64^a^	–	63.36 ± 35.54^c^	–
∆‐7‐avenasterol	–	–	60.13 ± 7.55^b^	–	81.87 ± 13.72^a^	–
Total sterols	3,946.03 ± 81.40^ef^	3,764.31 ± 101.46^ef^	4,095.11 ± 87.39^ef^	2,578.09 ± 80.83^f^	17,988.09 ± 1539.77^b^	7,545.94 ± 57.98^d^

Note

AS*, Annona senegalensis;* BL*, Brachystegia longifolia*; CD*, Caesalpinia decapetala*. DV*, Dodonaea viscosa;* EA, *Entada abyssinica;* II*, Ipomoea involucrate;* MA, Myrianthus *arboreus;* ME*, Maesopsis eminii;* PC*, Parinari curatellifolia;* ST*, Sterculia tragacantha;* TV*, Tephrosia vogelii;* UA*, Uvaria angolensis*

Data represent means ± *SD* (standard deviation) (*n* = 3). –: not identified. Values with different superscript letters ^(a‐i)^ within each row are significantly different at *p* < .05.

β‐sitosterol was the major component in all oil samples. Such trends have been reported in other unconventional oils such as watermelon (*Citrullus lanatus*), honeydew melon (*Cucumis melo*), sea buckthorn (*Hippophae rhamnoides*), red currant (*Ribes rubrum*), pomegranate (*Punica granatum*), Japanese quince (*Chaenomelesjaponica*), grape (*Vitis vinifera*), gooseberry (*Ribes uva‐crispa*), and apple (*Malus domestica*) (Górnaś & Rudzi, 2016). Low content was obtained in *S. tragacantha* oil (1,234.93 mg/kg), while two exceptional high contents were observed in *T.vogelii* oil (13,115.22 ± 122.61 mg/kg) and *B. longifolia* (16,564.81 ± 15.65 mg/kg). The β‐sitosterol of *B. longifolia* oil was twice that found in the oil of evening primrose (Phillips et al., 2002). The following important phytosterols contents were campesterol for some species and stigmasterol for others. The same pattern is also found in conventional oils as olive oils for stigmasterol (Poiana, 2012) and rapeseed oil for campesterol (Maniet et al., 2019). Thus, the campesterol amounts in the studied oils ranged between 120.25 ± 5.18 and 2,555.83 ± 1.96 mg/kg, respectively, for *M. eminii* and *B. longifolia* oils, whereas stigmasterol contribution ranged from 115.63 ± 0.51 mg/kg identified in *M. eminii* oil to 2,597.77 ± 13.17 mg/kg in *I. involucrate* oil. Except the campesterol, stigmasterol, and β‐sitosterol, all others sterols are found not to be detectable in all oil samples: for example, ∆‐7‐avenasterol and 24‐methylene‐cholesterol were identified in 3 types of oils. Cholesterol, which was not detected in *A. senegalensis* oil, ranged from 39.29 to 108.92 mg/kg in the oils extracted from the other species. These cholesterol contents are higher than those found in rapeseed and extra virgin olive oils (29 mg/kg) (Phillips et al., 2002), whereas they were lower than those reported in nutmeg oil (302.76 mg/kg) and white mustard oil (240.40 mg/kg) (Kozłowska et al., 2016). The order of cholesterol contents was as follows: *U. angolensis* > *S. tragacantha* > *P. curatellifolia* > *B. longifolia* oil but, without significant difference at *p* > .05. Another important sterol was found to be ∆‐5‐avenasterol. This phytosterol is reputed to have good antioxidant and antipolymerization activity in frying oils (White & Armstrong, 1986), it was identified in 10 oil samples. In *I. involucrate* and *U. angolensi s*oils, it was undetectable. The contents of ∆‐5‐avenasterol were in the following descending order: *T. vogelii* > *D. viscosa* > *I. involucrata* > *P. curatellifoli*a oil (*p* < .05). The lowest content was recorded in *D. viscosa* oil at 33.96 ± 4.17 mg/kg. Regarding phytostanols, campestanol was detected only in *B. longifolia* oil at 77.55 mg/kg whereas sitostanol was present in oils from 10 seed species and at relatively high contents. Thus, the highest sitostanol contents found ranged from 16,564.81 to 3,914.65 mg/ kg and order is as follows: *B. longifolia* > *T. vogelii* > *S. tragacantha* > *E. abyssinica* (*p* < .05). Contribution of ∆‐7‐stimasterol was found in oils extracted from 6 species. The highest content reached 383.53 mg/kg in *P. curatellifolia* oil, while the lowest was 31.13 mg/kg in *B. longifolia* oil. Low contributors to total phytosterols were provided by ∆‐5– 23‐stigmastadienol (<103.34 mg/kg), brassicasterol (<105 mg/kg oil), ∆‐clerosterol (<148.51), and 24‐stigmastadienol (<70.32 mg/kg). However, some of them showed appreciable contents in conventional oils. Brassicasterol was reported to reach a level of 792 mg/kg in cold pressed rapeseed oil (Tańska et al., 2017).

#### 3.3.2 Classification of the studied oils using HCA test

According to phytosterols contents, the HCA showed three different Clusters at the distance of 0.25 (Figure 1b). Cluster 1 was found to be formed by eight species segregated into two subclusters. The subcluster 1 contains only one specie (*B. longifolia*), which is characterized by its contents of β‐sitosterols, campesterol, cholesterol, and sitostanol that were significantly elevated (*p* < .05) in comparison with those of species in the subcluster 2. Cluster 2 gathered *P. curatellifoli*a and *D. viscose* oil. Their similarity was mainly due to the stigmasterol, ∆‐7‐campesterol, ∆‐7‐stigmasterol, and total sterol contents that showed relatively no significant differences (*p* > .05). Cluster 3 combined *I. involucrata* and *U. angolensis*. Although cholesterol, campesterol, stigmasterol, and β‐sitosterol contents of *I. involucrata* were significantly different (*p* < .05) from those of *U. angolensis*, the similarity of these two species strongly depends on them. Even though they were different, they were at the successive significance levels (Table 3).

### 3.4 Total phenol content (TPC)

After oil extraction with lipophilic solvent (hexane), many studies have shown the presence of polyphenols in oils (Górnaś et al., 2019; Kozłowska et al., 2016). Although polyphenols are known to be predominantly hydrophilic some of them are truly lipophilic and that is why they can be encountered in oils (Ginsburg et al., 2012; Peng et al., 2019). Furthermore, hydrophilic may be also carried along with the main substance being extracted.

**Figure 2:**
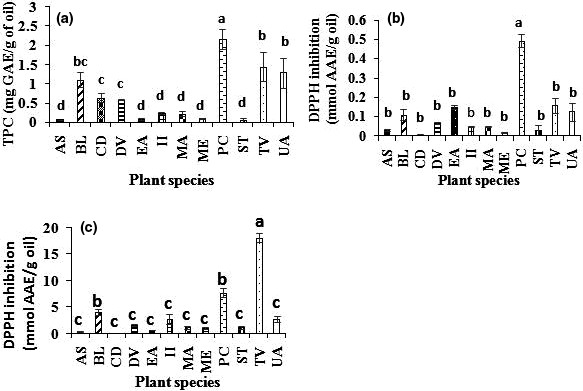
Total phenol content in methanolic extract (A), inhibition of DPPH with methanolic extract (B), and inhibition of DPPH with crude oil (C). PC, *Parinari curatellifolia*; MA, *Myrianthus arboreus*; ME, *Maesopsis eminii*; ST, *Sterculia tragacantha*; AS, *Annona senegalensis*; UA, *Uvaria angolensis*; CD, *Caesalpinia decapetala*. DV, *Dodonaea viscosa*; EA, *Entada abyssinica*; II, *Ipomoea involucrata*; TV, *Tephrosia vogelii*; BL, *Brachystegia longifolia*

Polyphenol content is an indication of a good oxidative stability and good quality of the oil. Polyphenols prevent the oxidation of unsaturated fatty acids (El‐beltagi & Mohamed, 2013) which may decrease the nutritional and sensory quality of oil. Polyphenols have been widely reported to be potent antioxidants. Furthermore, intake of such oil containing high levels of polyphenols improve human health. TPC of our oil samples is depicted in Figure 2a. According to Duncan test, TPCs were divided in 4 significantly different groups (*p* < .05). But overlaps were observed where one species could be found in two successive groups. The highest TPC was obtained in the group consisting of *P. curatellifolia* oil (2,160 ± 260 mg GAE/Kg oil) but slightly less than that found in the nutmeg oil (Kozłowska et al., 2016). The second group having relatively high TPC content was represented by three oils: *T. vogelii* > *U. angolensis* > *B. longifolia* with no significant difference at *p* > .05. Our results on TPC from these two groups were higher than those of virgin extra argan oil and quite similar to those found in virgin extra olive oil (Mirela & Apetrei, 2013; Seiquer et al., 2015), white mustard, coriander, and caraway (Kozłowska et al., 2016). However, extra virgin argan and extra virgin olive oils are characterized by the same important bioactivity owing to their polyphenol contents (Seiquer et al., 2015). One can expect significant antioxidant bioactivities from the first four species. The third group consisted of oils from three species, and their order was as follows: *B. longifolia* > *C. decapetala* > *D. viscosa* without significant differences (*p* > .05). The fourth group with low content gathered the TPC values of 6 species oils. The lowest (60 mg GAE/Kg oil) was found in *S. tragacantha* oil. However, these results were comparable to these reported in virgin argan oil (6–152 mg GAE/Kg) by (Martı & Mesı, 2011).

### 3.5 Antioxidant activities estimations using the DPPH assay

Estimations of the antioxidant activities of the twelve plant oils samples were performed using the DPPH assay and were expressed as mmol of ascorbic acid equivalent (AAE)/g oil as is shown in Figure 2b,c. The DPPH assay is one of the most used methods offering an evaluation of the antioxidant capacity (Shahidi & Zhong, 2015). Regarding the oil extracts antioxidant activities, values were ranged from 0.04 mmol up to 2.89 mmol AAE/g oil. However, based on Duncan's statistical test, almost all species had the same antioxidant activity. Antioxidant activity of *P. curatellifolia* was found to be statistically different (*p* < .05) compared to the eleven remaining species, whereas these latter ones were not significantly different (*p* > .05). Studies carried out on rice bran oil extract (Liu et al., 2019), cocoa, and olive oil extract (Bubonja‐sonje et al., 2011) reported antioxidant activities positively correlated with their polyphenol contents. With other antioxidant molecules such as tocopherols, they could extend the shelf life of oils and particularly those rich in polyunsaturated fatty acids.

According to the Duncan's test, antioxidant activities related to crude oils were classified into three groups of significant difference (*p* < .05). The group with the highest antioxidant activity consisted of one specie (*T. vogelii*), and it has reached 18.08 ± 7.08 mmol AAE/g oil. While the following group consisted of the antioxidant activities of *P. curatellifolia* and *B. longifolia* oils (with no significant difference at *p* > .05), the last group consists of nine species with not significantly different antioxidant activities (*p* > .05). The lowest antioxidant activity (0.04 ± 0.01 mmol AAE/g) was found analyzed in *C. decapetala* oil. Comparatively, the antioxidant activity evaluated in the methanolic extract was found to be clearly lower than that evaluated in the crude oil. This is because methanolic extracts contain only hydrophilic compounds, while the crude oil combines both the hyrophilic and lipophylic antioxidant compounds. The correlation coefficient between antioxidant activity and total polyphenol content was generally low in these two materials (methanolic extract and crude oil). The obtained correlation coefficient was higher between TPC and antioxidant activity of the extracts (r: 0.377) than that of TPC with the antioxidant activity of the oils (r: 0.283). This may suggest that the antioxidant activities of the oil may be due to other compounds (like tocopherols) not being extracted with polar solvent or to asynergy of phenolic compound with other compounds. Other researchers reported some lipophilic antioxidant compounds to more contribute for antioxidant activity than hydrophilic compounds (Martı & Mesı, 2011; Tuberoso et al., 2007).

## 4 CLASSIFICATION OF THE TWELVE OILS ACCORDING TO THE OVERALL STUDIED PARAMETERS

The HCA analysis of oil samples using all the determined parameters allowed segregating them in tree clusters (Figure 1c). Thus, the first consists of *I. involucrate, T. vogelii, A. senegalensis, U. angolensis, C. decapetala, and M. arboreus* oils. They are characterized by high contents of polyunsaturated fatty acid. While *M. arboreus* reached 89%, the others varied between 42% and 73%. Their similarity is also due to their high phytosterol contents. Cluster 2 consisted of the oil of a single species *(B. longifolia)*. This great distance observed in the species of *B. longifolia* compared to other oils could be due to its different parameters which are quite particular. It is characterized by Σ PUFAs and PUFA/ SFA significantly lower than those of other oils (*p* < .05). It also contains significantly (*p* < .05) higher content of phytosterols than other species oils. Cluster 3 gathered *E. abyssinica, M. eminii, D. viscosa, P. curatellifolia,* and *S. tragacantha* oil. Their similarities are attributed to the PUFA/ SFA and to their contents of phytosterols.

## 5 CONCLUSION

The obtained results show a wide range of important lipid compounds, some of which were characterized by great bioactivity. They were closely similar to the conventional oils content. Among 13 fatty acids detected, the major compounds for most species consisted of palmitic, oleic, linoleic, and stearic acids, while *P. curatellifolia* has exceptionally high amounts of erucic at 58.41 ± 0.77%. Furthermore, it was found that the studied oil plants were good sources of phytosterols. Their overall amounts ranged from 2,578 to 21,630.98 mg/kg and β‐sitosterol being the predominant in all seed oil extracts. Data on estimated antioxidant activity in methanolic extracts and crude oils revealed that in addition to the high TPC content, the studied crude oils may contain other bioactive compounds. These are probably the tocopherols and tocotrienols which could be the subject of further study. The analysis of the overall studied chemical composition of the twelve wild species showed that most of them can be considered as edible oils while others can be useful in cosmetic, pharmacy domains, and in different industrials applications.

## CONFLICT OF INTEREST

The authors declare no conflict of interest.
